# The HFpEF-ABA score predicts adverse cardiac remodelling and incident heart failure: a UK biobank study

**DOI:** 10.1093/eschf/xvag171

**Published:** 2026-07-07

**Authors:** Qingbo Shi, Yang Gao, Donghui Chen, Zhuocheng Shi, Chaowei Zhang, Yushuo Gu, Boyi Shi, Cao Ma, Zhiwen Zhang, Xingwei Wang, Yunxia Wang, Quan Guo, Bingchuan Geng, Muwei Li

**Affiliations:** Department of Cardiology, Central China Fuwai Hospital of Zhengzhou University, Fuwai Central China Cardiovascular Hospital, Zhengzhou, Henan 450000, China; Department of Cardiology, Central China Fuwai Hospital of Zhengzhou University, Fuwai Central China Cardiovascular Hospital, Zhengzhou, Henan 450000, China; Department of Cardiology, Zhengzhou University People’s Hospital, Zhengzhou, Henan 450000, China; Department of Cardiology, Central China Fuwai Hospital of Zhengzhou University, Fuwai Central China Cardiovascular Hospital, Zhengzhou, Henan 450000, China; Department of Cardiology, Central China Fuwai Hospital of Zhengzhou University, Fuwai Central China Cardiovascular Hospital, Zhengzhou, Henan 450000, China; Department of Cardiology, Central China Fuwai Hospital of Zhengzhou University, Fuwai Central China Cardiovascular Hospital, Zhengzhou, Henan 450000, China; Department of Cardiology, Zhengzhou University People’s Hospital, Zhengzhou, Henan 450000, China; Department of Cardiology, Central China Fuwai Hospital of Zhengzhou University, Fuwai Central China Cardiovascular Hospital, Zhengzhou, Henan 450000, China; The First Clinical School of Zhengzhou University, Zhengzhou, Henan 450000, China; Department of Cardiology, Central China Fuwai Hospital of Zhengzhou University, Fuwai Central China Cardiovascular Hospital, Zhengzhou, Henan 450000, China; Department of Cardiology, Central China Fuwai Hospital of Zhengzhou University, Fuwai Central China Cardiovascular Hospital, Zhengzhou, Henan 450000, China; Department of Cardiology, Central China Fuwai Hospital of Zhengzhou University, Fuwai Central China Cardiovascular Hospital, Zhengzhou, Henan 450000, China; Department of Radiology, Zhengzhou Central Hospital Affiliated to Zhengzhou University, Zhengzhou, Henan 450000, China; Department of Cardiology, Central China Fuwai Hospital of Zhengzhou University, Fuwai Central China Cardiovascular Hospital, Zhengzhou, Henan 450000, China; Department of Cardiology, Central China Fuwai Hospital of Zhengzhou University, Fuwai Central China Cardiovascular Hospital, Zhengzhou, Henan 450000, China; Henan Provincial Clinical Research Center for Cardiovascular Disease, Zhengzhou, Henan 450000, China; Department of Cardiology, Central China Fuwai Hospital of Zhengzhou University, Fuwai Central China Cardiovascular Hospital, Zhengzhou, Henan 450000, China; Department of Cardiology, Zhengzhou University People’s Hospital, Zhengzhou, Henan 450000, China; Henan Provincial Clinical Research Center for Cardiovascular Disease, Zhengzhou, Henan 450000, China; Central China Subcenter of National Center for Cardiovascular Diseases, Henan Cardiovascular Disease Center, Zhengzhou, Henan 450000, China

**Keywords:** HFpEF-ABA score, Heart failure, Preventive screening, Cardiac magnetic resonance, Risk stratification

## Abstract

**Introduction:**

The HFpEF-ABA score (age, body mass index, atrial fibrillation) was originally derived to diagnose heart failure with preserved ejection fraction (HFpEF) in symptomatic patients. However, its utility as a preventive screening tool to identify preclinical risk in unselected community populations remains unvalidated.

To validate the HFpEF-ABA score in a large community-based cohort free of heart failure and structural heart disease, utilizing a dual validation approach combining cardiac magnetic resonance phenotyping and long-term outcome prediction.

**Methods:**

We analysed 416 374 UK Biobank participants without baseline heart failure. Participants were stratified by HFpEF-ABA score into low, intermediate, and high risk. We assessed biological validity in a subcohort of 65 293 participants utilizing cardiac magnetic resonance, and clinical validity via prospective adjudication of heart failure hospitalization over a median follow-up of 14.9 years.

**Results:**

Structurally, elevated scores identified characteristic patterns of adverse cardiac remodelling in individuals at high risk for incident heart failure despite the absence of a clinical diagnosis: high-risk individuals exhibited profound left atrial dysfunction (LA ejection fraction 55.48% vs. 61.92%, *P* < .001) and distinct concentric ventricular remodelling compared with low-risk participants. Clinically, this structural substrate translated into a 6.4-fold excess risk of incident heart failure hospitalization (hazard ratio [HR] 6.44, 95% confidence interval [CI] 5.86–7.08, *P* < .001) and a 3.5-fold increased risk of the composite outcome (*P* < .001). The score demonstrated robust discrimination, outperforming its individual components.

**Conclusion:**

The HFpEF-ABA score demonstrates significant prognostic value for identifying individuals at high risk for incident heart failure and is associated with adverse cardiac remodelling and subclinical cardiac changes. These findings support repositioning the score from a diagnostic aid to a population-level screening instrument, supporting earlier identification of high-risk individuals before overt clinical heart failure develops.

## Graphical Abstract

**Graphical Abstract:**
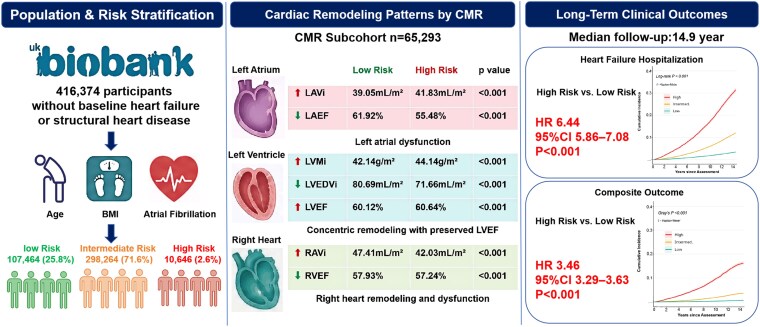

## Introduction

Heart failure with preserved ejection fraction (HFpEF) accounts for approximately half of all heart failure cases, imposing a burden of morbidity and mortality comparable with that of heart failure with reduced ejection fraction (HFrEF).^1^ While recent therapeutic breakthroughs, including SGLT2 inhibitors and GLP-1 receptor agonists, offer new avenues for management, their impact is limited by significant underdiagnosis.^2,3^ It is estimated that up to one-third of adults presenting with exertional dyspnoea have unrecognized HFpEF, often diagnosed only after overt clinical decompensation.^4^

To shift the paradigm from reactive treatment to active prevention, early identification of individuals at high risk for incident heart failure is crucial. However, widespread screening in primary care remains challenging. Current diagnostic pathways, such as the HFA-PEFF algorithm, rely heavily on echocardiography and natriuretic peptides.^5^ These data are rarely available during routine primary care encounters, creating a critical unmet need for scalable, data-driven screening tools that utilize readily available clinical variables.

The HFpEF-ABA score, a parsimonious model incorporating only age, body mass index (BMI), and atrial fibrillation status, was recently developed to address this need.^6^ While the score demonstrated robust accuracy (area under the curve [AUC] 0.813–0.839) for diagnosing HFpEF in symptomatic patients, its potential utility as an upstream screening instrument in the general population remains unexplored. Specifically, whether this simple clinical score correlates with the subclinical structural substrate of HFpEF in asymptomatic individuals is unknown.

The UK Biobank offers a unique platform to bridge this gap, featuring extensive phenotypic data and cardiac magnetic resonance (CMR) imaging in a large community-based cohort.^7^ In this study, we aimed to provide a dual validation of the HFpEF-ABA score in individuals free of diagnosed heart failure: (i) biologically, by characterizing its association with quantitative CMR structural and functional phenotypes to determine if it captures remodelling patterns associated with HFpEF; and (ii) clinically, by evaluating its ability to predict long-term incident heart failure and mortality. We hypothesized that the HFpEF-ABA score would identify structural remodelling patterns associated with increased risk of heart failure, characterized by left atrial dysfunction and concentric remodelling, and effectively stratify risk for future adverse outcomes.

## Methods

### Study population and design

The UK Biobank is a prospective population-based cohort study recruited between 2006 and 2010 from across the UK.^8^ All participants provided written informed consent. The study was conducted under UK Biobank approval and Research Ethics Committee approval (REC 11/NW/0382).

**Figure 1:**
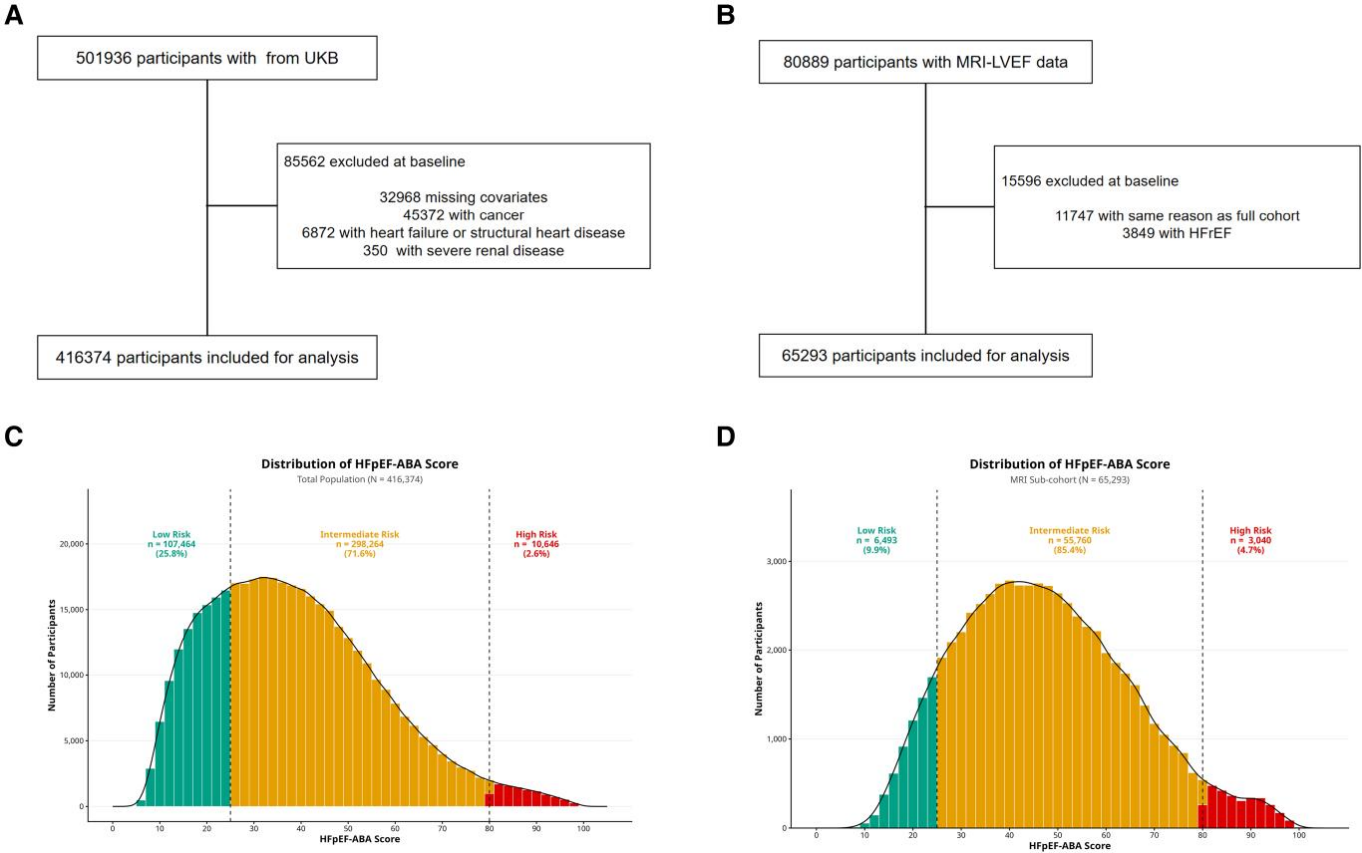
Study flowcharts and distribution of the HFpEF-ABA score. (A) Selection of the full cohort from the UK Biobank. (B) Selection of the subcohort with cardiac MRI data. (C–D) Histograms showing the distribution of HFpEF-ABA scores in the full cohort (left) and MRI subcohort (right). Risk categories: low (<25%), intermediate (25–80%), and high (>80%)

**Primary cohort**: from 501 936 participants with complete baseline data, we excluded 85 562 participants due to missing covariates (*n* = 32 968), prevalent cancer (*n* = 45 372), pre-existing heart failure, or structural heart disease including cardiomyopathies, severe valvular disease, congenital heart disease, or pericardial disease (*n* = 6872), and severe chronic kidney disease (*n* = 350). This yielded a final analytical cohort of 416 374 participants (*Figure 1A*).

**CMR subcohort**: from 80 889 participants who underwent CMR imaging (predominantly at the imaging visit occurring 2009–2013), we applied identical exclusion criteria plus additional exclusion for left ventricular ejection fraction (LVEF) <50%, yielding 65 293 participants for cardiac structure–function analyses (*Figure 1B*).

### HFpEF-ABA score calculation

The HFpEF-ABA probability was calculated using the validated logistic regression equation:


Logodds=−7.788751+(0.062564×age)+(0.135149×BMI)+(2.040806×AF)

Where AF is coded as 1 if present (paroxysmal or persistent/permanent atrial fibrillation or atrial flutter) and 0 if absent. The predicted probability of HFpEF was then derived as: Probability (%) = 100/[1 + e^ (−log odds)]

Participants were stratified into three risk categories: low risk (<25%), intermediate risk (25–80%), and high risk (>80%).

### Baseline assessments

#### Socio-demographic and anthropometric assessment

Baseline characteristics, including age, sex, ethnicity, education level, and socio-economic status (Townsend Deprivation Index), were obtained via standardized touch-screen questionnaires. Lifestyle factors included smoking status, alcohol consumption, and physical activity, quantified as metabolic equivalent of task (MET)-minutes per week. Anthropometric measurements included BMI, calculated as weight in kilograms divided by height in metres squared. For CMR indexing, body surface area (BSA) was derived using the Mosteller formula.

#### Comorbidities, medications, and biochemistry

Comorbidities were ascertained using a composite of self-reported physician diagnoses and linked hospital admission records (International Classification of Diseases, 10th Revision [ICD-10]). Specific conditions included hypertension (I10-I15), diabetes mellitus (E10-E14), coronary artery disease (I20-I25), and atrial fibrillation (I48), ensuring diagnoses preceded the baseline or CMR assessment. Medication use was categorized into antihypertensive agents, lipid-lowering drugs (statins), antidiabetic agents, and antiplatelet therapies based on verified nurse interviews. Laboratory profiling included C-reactive protein (CRP), low-density lipoprotein (LDL) cholesterol, and HbA1c. Renal function was assessed using the estimated glomerular filtration rate (eGFR), calculated via the 2021 CKD-EPI creatinine–cystatin C equation.^9^

### Cardiac magnetic resonance imaging

CMR was performed on 1.5 Tesla scanners (MAGNETOM Aera, Syngo Platform VD13A, Siemens Healthcare, Erlangen, Germany) using standardized protocols.^10^ Biventricular volumes, mass, and function were quantified from balanced steady-state free precession cine images. Left atrial maximum volume (LAVmax) and right atrial maximum volume (RAVmax) were measured at ventricular end-systole. Atrial ejection fractions were calculated as: (maximum volume − minimum volume)/maximum volume × 100%. Global longitudinal strain (GLS) was derived from feature-tracking analysis of standard long-axis cine images.

All CMR parameters were indexed to body surface area except ejection fractions and GLS. Measurements were performed using validated automated segmentation algorithms with manual quality control.

### Clinical outcomes

The primary outcomes were: (i) heart failure hospitalization, defined as first hospital admission with primary ICD-10 diagnosis code I50 or I11.0; (ii) composite outcome of heart failure hospitalization or all-cause mortality; and (iii) all-cause mortality. Death dates were ascertained through linkage with the National Health Service Information Centre (England and Wales) and NHS Central Register Scotland. Follow-up extended from baseline assessment through 31 December 2023, with median follow-up 14.94 years (interquartile range 14.25–15.61 years).

### Statistical analysis

#### Descriptive statistics and structural phenotyping

Descriptive statistics and structural phenotyping continuous variables are presented as medians (interquartile range [(IQR]) and compared using Kruskal–Wallis tests due to non-normal distributions. Categorical variables are reported as frequencies (percentages) and compared using χ2 tests. To evaluate the biological association between the HFpEF-ABA score and cardiac remodelling, we assessed differences in CMR parameters across risk categories using Wilcoxon rank-sum tests. Additionally, we explored non-linear trends between the continuous score and structural phenotypes using generalized additive models (GAM) with LOESS smoothing and Spearman correlation coefficients.

To confirm that the associations between HFpEF-ABA risk category and CMR parameters were independent of age, we performed multivariable linear regression in the CMR subcohort, with the low risk category as reference. Model 1 adjusted for age and sex; Model 2 (fully adjusted) additionally adjusted for BMI, hypertension, diabetes mellitus, and coronary artery disease. Results are reported as β coefficients with 95% confidence intervals (CIs).

#### Survival analysis and risk stratification

We estimated hazard ratios (HRs) and 95% CIs for clinical outcomes using Cox proportional hazards models. Multivariable analyses were performed using three progressive adjustment models: Model 1 adjusted for demographics (sex, ethnicity, education, Townsend deprivation index); Model 2 added lifestyle factors (smoking, alcohol intake, physical activity); and Model 3 further adjusted for comorbidities and medications (hypertension, diabetes, coronary artery disease, eGFR, and use of lipid-lowering, antihypertensive, antidiabetic, or antiplatelet agents). The proportional hazards assumption was verified using Schoenfeld residuals. To account for the competing risk of death, Fine–Gray subdistribution hazard models were employed for the analysis of heart failure hospitalization. Restricted cubic splines (RCS) with three knots were used to visualize the dose–response relationship between the continuous HFpEF-ABA probability and incident risk.^11^

#### Predictive performance and robustness

Discriminative ability was evaluated using Harrell’s C-index and time-dependent receiver operating characteristic (ROC) curves (10-year horizon). Calibration was assessed by comparing predicted vs. observed risks across deciles. Clinical utility was determined via decision curve analysis (DCA).^12^ To ensure robustness, we performed landmark analyses excluding events within the first 2 years to mitigate reverse causality. Stratified analyses were conducted across key subgroups (age, sex, BMI, comorbidities), with heterogeneity assessed using likelihood ratio tests for interaction.

To formally evaluate the incremental predictive value of the HFpEF-ABA score beyond age alone, we compared C-indices using z-tests and calculated the Integrated Discrimination Improvement (IDI) and continuous Net Reclassification Improvement (NRI) at the 5-year horizon (survIDINRI package), comparing an age-only baseline model against an augmented model incorporating the HFpEF-ABA probability. These analyses were conducted as sensitivity analyses.

All analyses were performed using R version 4.3.1. A two-sided *P*-value <.05 was considered statistically significant.

## Results

### Baseline characteristics

Full cohort: among 416 374 participants (median age 58 years, 54% women, 94% White ethnicity), the HFpEF-ABA score distribution yielded 107 464 (25.8%) low-risk, 298 264 (71.6%) intermediate-risk, and 10 646 (2.6%) high-risk individuals (*Figure 1C*). High-risk participants were older (median 64 vs. 48 years), more obese (BMI 39.3 vs. 23.6 kg/m^2^) (*Table 1*). High-risk individuals exhibited greater prevalence of hypertension (64% vs. 9%), diabetes mellitus (21% vs. 1%), and coronary artery disease (18% vs. 1%), along with reduced eGFR (79.0 vs. 101.0 ml/min/1.73 m^2^) and elevated inflammatory markers (CRP 3.43 vs. 0.74 mg/L) (all *P* < .001).

**Table 1:** Baseline clinical characteristics of the study population stratified by HFpEF-ABA risk categories

Characteristic	Overall (*n* = 416 374)	Low (<25%) (*n* = 107 464)	Intermediate (25–80%) (*n* = 298 264)	High (>80%) (*n* = 10 646)	*P*
Age	58 [50, 63]	48 [44, 52]	61 [55, 65]	64.00 [60.00, 67.00]	<.001
BMI	26.72 [24.13, 29.86]	23.56 [21.79, 25.44]	27.89 [25.44, 30.78]	39.34 [31.59, 43.29]	<.001
Male sex	193024 (46.4)	41834 (38.9)	146198 (49.0)	4992 (46.9)	<.001
Ethnicity					<.001
White	392085 (94.2)	98977 (92.1)	282928 (94.9)	10180 (95.6)	
Asian	8407 (2.0)	2996 (2.8)	5330 (1.8)	81 (0.8)	
Black	6679 (1.6)	1907 (1.8)	4555 (1.5)	217 (2.0)	
Townsend	−2.15 [−3.65, 0.50]	−1.99 [−3.60, 0.77]	−2.23 [−3.68, 0.35]	−1.41 [−3.26, 1.77]	<.001
Education (college degree)	47807 (11.5)	15140 (14.1)	31827 (10.7)	840 (7.9)	<.001
Smoking					<.001
Never	229345 (55.3)	66152 (61.7)	158142 (53.2)	5051 (47.8)	
Previous	141519 (34.1)	26539 (24.8)	110198 (37.1)	4782 (45.3)	
Current	43894 (10.6)	14479 (13.5)	28686 (9.7)	729 (6.9)	
Alcohol consumption					<.001
Never	18143 (4.4)	4385 (4.1)	12996 (4.4)	762 (7.2)	
Previous	14317 (3.4)	3168 (3.0)	10447 (3.5)	702 (6.6)	
Current	383330 (92.2)	99735 (93.0)	274430 (92.1)	9165 (86.2)	
Physical activity ≥600 MET-min/week	264892 (63.6)	73870 (68.7)	185902 (62.3)	5120 (48.1)	<.001
Hypertension	110562 (26.6)	9558 (8.9)	94248 (31.6)	6756 (63.5)	<.001
Diabetes mellitus	20451 (4.9)	1254 (1.2)	16999 (5.7)	2198 (20.6)	<.001
Coronary artery disease	19547 (4.7)	933 (0.9)	16693 (5.6)	1921 (18.0)	<.001
Atrial fibrillation	4647 (1.1)	0 (0.0)	839 (0.3)	3808 (35.8)	<.001
Antihypertensive agents	75268 (18.1)	4736 (4.4)	64641 (21.7)	5891 (55.3)	<.001
Lipid-lowering agents	61414 (14.7)	3061 (2.8)	54025 (18.1)	4328 (40.7)	<.001
Antidiabetic agents	14587 (3.5)	1002 (0.9)	11969 (4.0)	1616 (15.2)	<.001
Antiplatelet agents	2458 (0.6)	133 (0.1)	2132 (0.7)	193 (1.8)	<.001
eGFR, ml/min/1.73 m^2^	91.35 [81.65, 100.44]	100.97 [92.94, 107.91]	88.32 [79.38, 96.78]	78.99 [69.17, 88.16]	<.001
C-reactive protein, mg/l	1.31 [0.65, 2.71]	0.74 [0.40, 1.51]	1.53 [0.79, 3.02]	3.43 [1.64, 6.99]	<.001
LDL cholesterol, mmollL	3.52 [2.96, 4.12]	3.34 [2.86, 3.88]	3.61 [3.02, 4.20]	3.16 [2.57, 3.84]	<.001
HbA1c, mmol/mol	35.20 [32.70, 37.80]	33.50 [31.30, 35.80]	35.70 [33.30, 38.30]	38.40 [35.30, 43.30]	<.001

Abbreviations: BMI, body mass index; MET, metabolic equivalent of task; eGFR, estimated glomerular filtration rate; CRP, C-reactive protein; LDL, low-density lipoprotein; HbA1c, glycated haemoglobin; IQR, interquartile range

CMR subcohort: The 65 293 participants undergoing CMR (median age 66 years, 54% women) demonstrated similar risk stratification: 6493 (9.9%) low risk, 55 760 (85.4%) intermediate risk, and 3040 (4.7%) high risk (*Figure 1D*). Compared with the full cohort, CMR participants were slightly older and healthier (lower smoking rates, higher education), reflecting the volunteer nature of the imaging substudy.

### HFpEF-ABA score and cardiac structure–function

**Figure 2:**
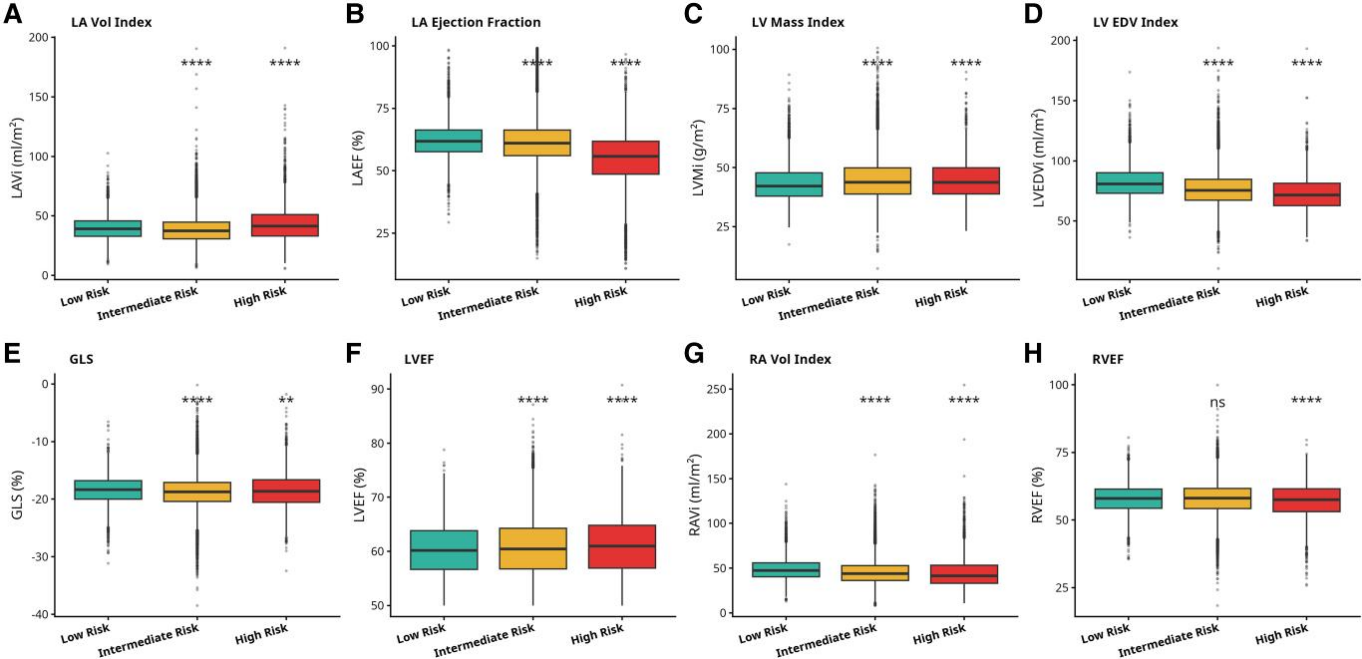
Comparison of cardiac structural and functional indices across risk groups. Boxplots showing cardiac magnetic resonance (CMR) parameters among low, intermediate, and high-risk groups. (A–B) Left atrial volume index and ejection fraction. (C–D) Left ventricular mass index and end-diastolic volume index. (E–F) Global longitudinal strain and LV ejection fraction. (G–H) Right atrial volume index and RV ejection fraction. **** *P* < .0001 compared with the low-risk group

**Figure 3:**
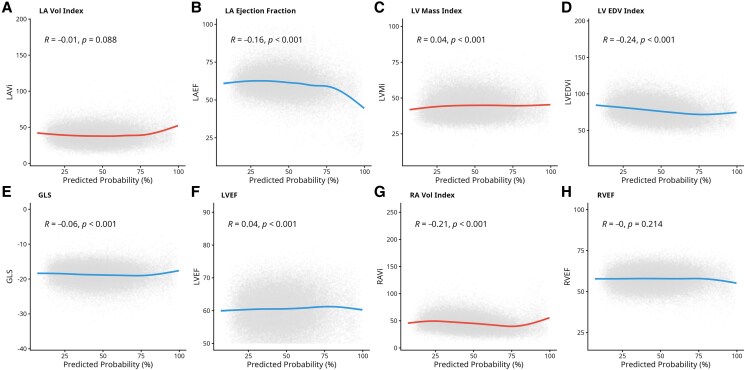
Continuous association between HFpEF-ABA probability and cardiac remodelling. Scatterplots with smooth fitted curves showing the correlations between predicted HFpEF probability and CMR indices. R indicates the Spearman correlation coefficient.

Atrial remodelling: higher HFpEF-ABA scores were strongly associated with adverse left atrial remodelling (*Figure 2*). Compared with low-risk individuals, high-risk participants demonstrated increased left atrial volume index (41.83 vs. 39.05 ml/m^2^, *P* < .001) and markedly reduced LA ejection fraction (55.48% vs. 61.92%, *P* < .001), indicating impaired atrial reservoir and contractile function. These categorical differences were corroborated by continuous associations showing progressive LA enlargement (Spearman *r* = −0.01, *P* = .088) and functional deterioration (*r* = −0.16, *P* < .001) with increasing HFpEF-ABA probability (Supplementary Table S1, *Figure 3*).

Ventricular remodelling: high-risk individuals exhibited concentric remodelling patterns with increased LV mass index (44.14 vs. 42.14 g/m^2^, *P* < .001) despite modestly smaller LV end-diastolic volume index (71.66 vs. 80.69 ml/m^2^, *P* < .001) (*Figure 2*). This discordant chamber size-to-mass relationship suggests diastolic dysfunction and reduced ventricular compliance characteristic of HFpEF. LVEF demonstrated a J-shaped relationship with HFpEF-ABA probability, with high-risk individuals showing a modestly higher mean LVEF (60.64% vs. 60.12%, *P* < .001). This finding aligns with prior observations of LVEF distribution patterns in HFpEF across its spectrum.^13^

Global longitudinal strain analysis revealed a biphasic association: preserved or augmented strain at lower risk scores transitioning to mild reduction at the highest risk levels (*P* < .001 across groups), suggesting subtle systolic dysfunction may emerge in advanced preclinical stages.

Right heart remodelling: high-risk status was associated with right atrial enlargement (RAVi: 42.03 vs. 47.41 ml/m^2^, *P* < .001) and reduced RV ejection fraction (57.24% vs. 57.93%, *P* < .001), indicating secondary right heart involvement even in the absence of overt HFpEF.

After multivariable adjustment for age, sex, BMI, hypertension, diabetes mellitus, and coronary artery disease, the associations between high risk category and impaired LA ejection fraction (*β* = −4.61%, 95% CI −5.10 to −4.11%, *P* < .001) and elevated LA volume index (*β* = + 5.39 ml/m^2^, 95% CI 4.76–6.02, *P* < .001) remained highly significant, confirming these structural abnormalities are not attributable to age alone (Supplementary Table S2).

### Clinical outcomes

During a median follow-up of 14.9 years, 13 319 participants (3.2%) experienced heart failure hospitalization, 37 250 (8.9%) died, and 45 272 (10.9%) experienced the composite outcome.

**Figure 4:**
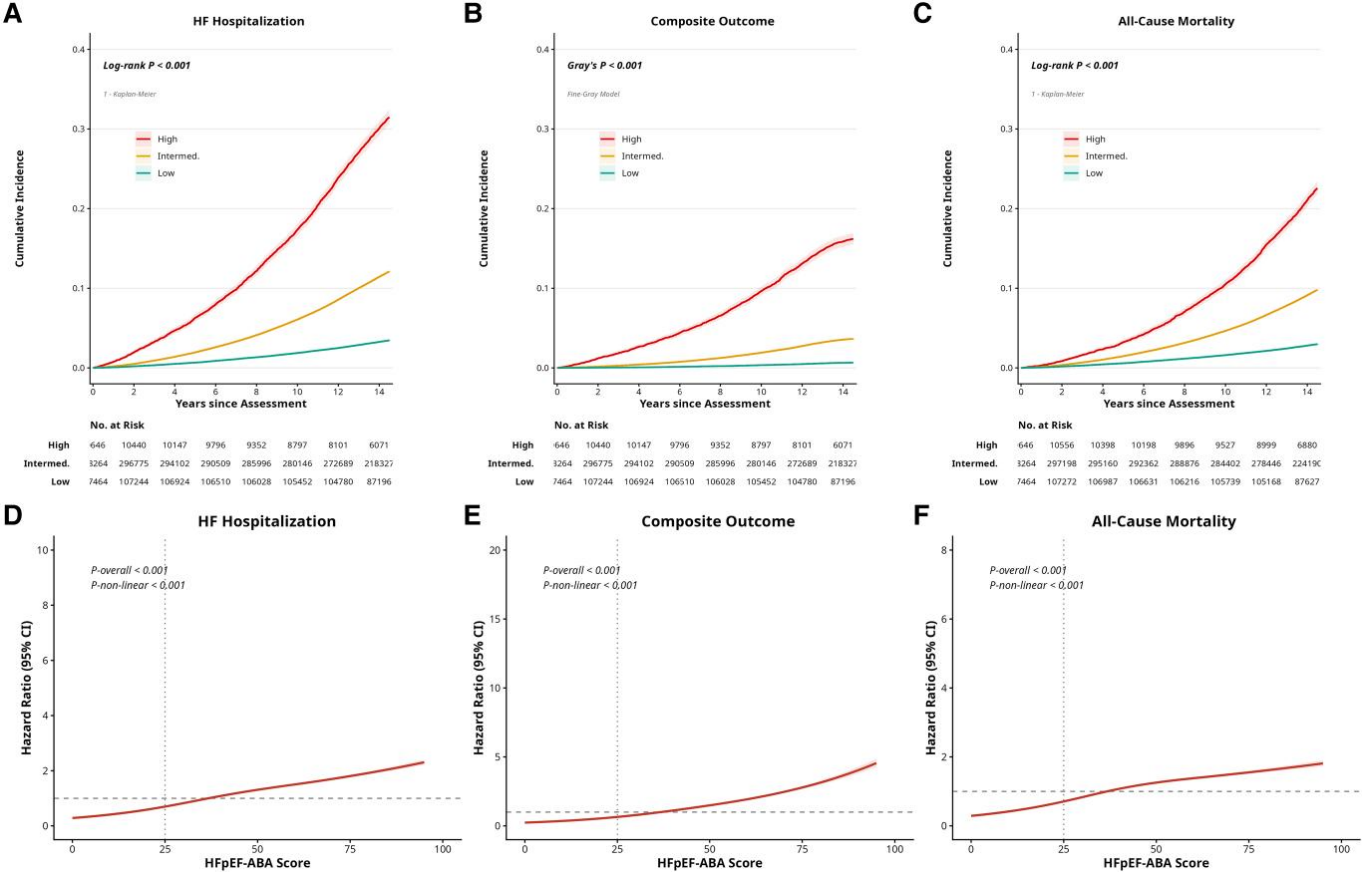
Cumulative incidence and dose–response relationships of the HFpEF-ABA score with adverse outcomes. (A–C) Cumulative incidence curves stratified by HFpEF-ABA risk groups (low, intermediate, and high). (A) HF hospitalization, estimated using the cumulative incidence function accounting for the competing risk of death (Fine–Gray model). (B) The primary composite outcome (first occurrence of HF hospitalization or all-cause mortality), estimated using the Kaplan–Meier method. (C) All-cause mortality, estimated using the Kaplan–Meier method. (D–F) Restricted cubic spline analyses illustrating the multivariable-adjusted hazard ratios (solid red lines) and 95% confidence intervals (shaded areas) for the association between the continuous HFpEF-ABA score and adverse outcomes. (D) HF hospitalization. (E) Composite outcome. (F) All-cause mortality

Risk stratification by HFpEF-ABA categories, the HFpEF-ABA score demonstrated robust risk stratification across all end-points. Kaplan–Meier analysis showed early and progressive divergence in event rates (Log-rank *P* < .001; *Figure 4A–C*). For heart failure hospitalization, the cumulative incidence at 14 years showed a striking gradient: 3.5% in the low-risk group, rising to 12.6% in the intermediate-risk group, and reaching 31.7% in the high-risk group.

**Figure 5:**
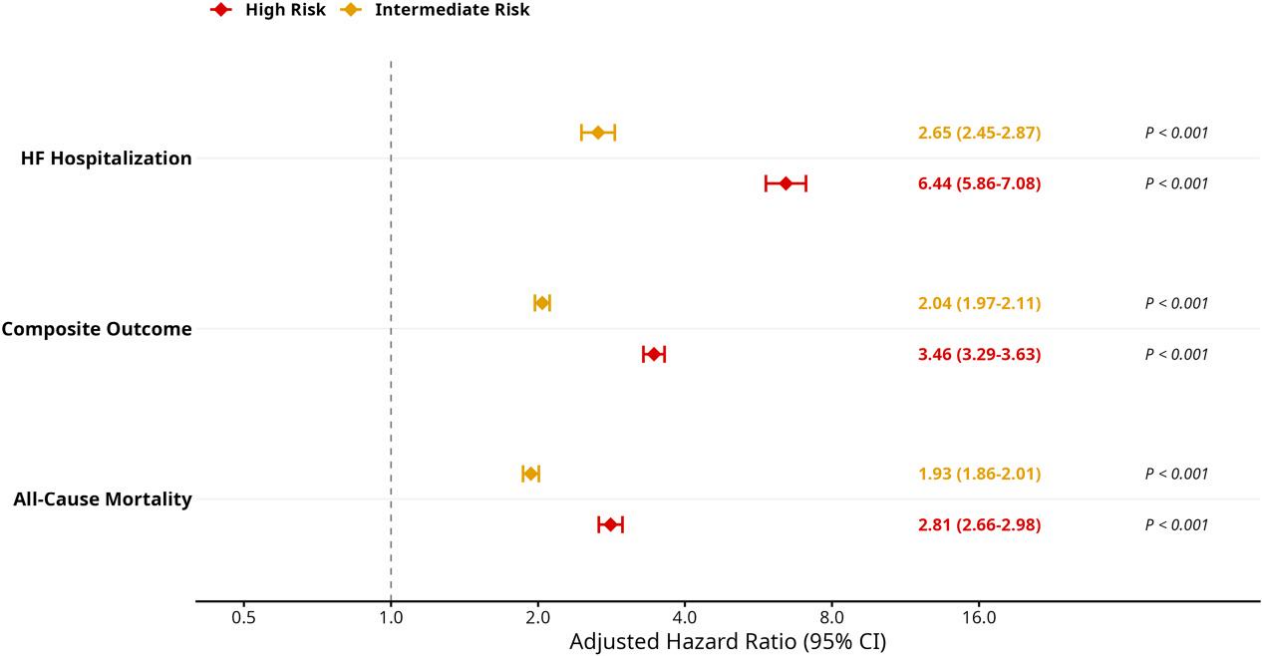
Adjusted hazard ratios for adverse outcomes by risk group. Forest plot showing the multivariable-adjusted hazard ratios (HR) for HF hospitalization, composite outcome, and all-cause mortality. The Low Risk group serves as the reference. Models were adjusted for all covariates (Model 3)

In fully adjusted models (*Figure 5*, Supplementary Table S3), compared with low-risk participants, intermediate-risk individuals had a 2.65-fold risk (HR 2.65, 95% CI 2.45–2.87), while high-risk individuals faced a 6.44-fold excess risk (HR 6.44, 95% CI 5.86–7.08, *P* < .001).Similarly, for all-cause mortality and the composite outcome, high-risk individuals exhibited significantly elevated risks (HR 2.81 [2.66–2.98] and HR 3.46 [3.29–3.63], respectively) compared with the low-risk reference group, independent of traditional cardiovascular risk factors.

Restricted cubic splines analyses visualized the continuous association between HFpEF-ABA probability and clinical outcomes (*Figure 4D–F*). There was a significant non-linear, monotonic increase in risk for all end-points as the score increased (*P* non-linear < .001). The risk of heart failure hospitalization escalated sharply at higher score ranges, confirming the score’s ability to identify individuals at the highest trajectory of risk.

### Subgroup and sensitivity analyses

Landmark analysis: After excluding events within the first 2 years of follow-up (*n* = 414 459 remaining), associations remained robust with minimal attenuation of effect sizes (Supplementary Figure S1, Supplementary Table S4), suggesting reverse causality does not substantially explain observed relationships. High-risk participants maintained 6.5-fold excess heart failure risk (HR 6.45, 95% CI 5.86–7.11) and 2.9-fold mortality excess (HR 2.88, 95% CI 2.72–3.05) compared with low-risk individuals.

Subgroup analyses: The association between high HFpEF-ABA risk and composite outcomes was consistent across prespecified subgroups (Supplementary Figure S2, Supplementary Table S5), with statistically significant interactions observed for age (stronger in younger participants, *P* < .001), diabetes (stronger in non-diabetic, *P* < .001), chronic kidney disease (stronger in preserved renal function, *P* < .001), smoking status (stronger in non-/ex-smokers, *P* < .001), and coronary artery disease (stronger without prevalent CAD, *P* < .001). These interaction patterns suggest the HFpEF-ABA score may be particularly informative for risk stratification in individuals without advanced comorbidities, where traditional cardiovascular risk assessment may underestimate HFpEF-related risk.

### Predictive performance

Discrimination: the HFpEF-ABA score demonstrated good discrimination for 10-year heart failure hospitalization (C-index 0.749, 95% CI 0.743–0.755), substantially outperforming individual components: age alone (C-index 0.730), BMI alone (0.642), or atrial fibrillation alone (0.533) (Supplementary Figure S3A). Time-dependent ROC analysis confirmed consistent discriminative ability across follow-up duration.

Clinical utility: DCA showed net benefit of the HFpEF-ABA score over treat-all or treat-none strategies across clinically relevant threshold probabilities from 2 to 15% (Supplementary Figure S3B). Compared with individual components, the integrated score provided superior net benefit across all decision thresholds, with maximal incremental benefit observed at 5–8% threshold probabilities.

Calibration: the HFpEF-ABA score demonstrated excellent calibration between predicted and observed 10-year heart failure risk across deciles of predicted probability (Supplementary Figure S3C). Visual inspection of the calibration plot revealed that the HFpEF-ABA score closely aligned with the line of perfect calibration, showing minimal deviation. Notably, the ABA score maintained robust calibration accuracy comparable with the age-only model while offering superior discrimination. Calibration remained robust in sensitivity analyses stratified by sex, age groups, and BMI categories.

As a sensitivity analysis, the HFpEF-ABA score demonstrated significantly superior discrimination for HF hospitalization compared with age alone (ΔC = + 0.019, *P* < .001), with significant incremental reclassification improvement (IDI 0.37%, *P* < .001; NRI 32.26%, *P* < .001), confirming meaningful predictive value beyond age (Supplementary Table S6).

## Discussion

Our study expands the utility of the HFpEF-ABA score from a diagnostic aid to a preventive screening instrument through its validation in 416 374 community participants free of heart failure and structural heart disease. While originally derived to diagnose established disease in symptomatic patients (AUC 0.839), we demonstrate its critical value in detecting early risk within this unselected population.^6^ Biologically, in a nested subcohort of 65 293 individuals, elevated scores strongly associated with the structural remodelling features associated with increased risk of heart failure, specifically significant left atrial dysfunction and concentric remodelling, confirming the identification of measurable pathophysiology even in the absence of a diagnosis.^14^ Clinically, this substrate translated into a 6.4-fold excess risk of heart failure hospitalization over 14.94 years (*P* < .001). This convergence of subclinical phenotyping and long-term prognosis establishes the score as a viable tool for population-level stratification, supporting a paradigm shift from reactive diagnosis to the active early identification and management of individuals at high risk for incident heart failure before irreversible damage occurs.

It is important to acknowledge that age is the primary mathematical driver of the score’s predictive power. However, our goal was not to derive a novel mathematical model to beat ‘age alone’, but rather to test the opportunistic utility of an already established diagnostic tool for upstream risk stratification. Incorporating BMI and AF retains the specific pathophysiological drivers of the HFpEF phenotype, allowing for a unified ‘red-flag’ system from primary care to specialized diagnosis. Formal sensitivity analyses confirmed significant incremental discrimination beyond age alone for HF hospitalization.

### Structural validation and biological plausibility

A pivotal finding of our study is that the HFpEF-ABA score, originally derived from clinical parameters, successfully identifies the structural remodelling features associated with HFpEF in undiagnosed individuals.^15^ This confirms that the score captures early pathophysiological changes associated with heart failure development years before clinical decompensation.

Left atrial myopathy as a cumulative barometer. The most pronounced phenotype in high-risk individuals was profound left atrial dysfunction. The observation of significantly impaired left atrial ejection fraction (LAEF) and increased left atrial volume index is clinically momentous.^16–18^ In the PARAMOUNT trial, the reduction of LA volume was a key marker of therapeutic success with sacubitril/valsarta.^19^ Our data suggest that the HFpEF-ABA score identifies individuals who have already developed the pathological substrate, atrial myopathy, that therapeutic agents aim to reverse. This confirms the LA’s role as a physiological ‘barometer’ of distinct, cumulative diastolic stress, often detectable before overt ventricular stiffening.^20,21^

The ‘small, stiff heart’ phenotype. Importantly, the observed atrial dysfunction was accompanied by a ventricular remodelling pattern characterized by concentric remodelling, increased LV mass, and smaller ventricular volumes. High-risk individuals maintained preserved LVEF despite these structural changes. This pattern is consistent with the classic ‘small, stiff heart’ phenotype commonly observed in HFpEF, where LVEF may remain preserved despite impaired ventricular compliance and elevated filling pressures.^13,22^ By distinguishing this concentric/hyperdynamic trajectory from the eccentric remodelling typical of pre-HFrEF, our score demonstrates specific phenotypic precision.^23^ Furthermore, if subtle reductions in GLS were present, this would align with findings from the PARAMOUNT imaging substudy, confirming latent systolic dysfunction despite preserved LVEF.^24–26^

Chronicity and integrated risk. Finally, the association with secondary right ventricular remodelling implies that elevated left-sided filling pressures have been sufficiently chronic to induce backward pulmonary vascular consequence.^27,28^ Importantly, these structural associations persisted after rigorous adjustment for comorbidities. This demonstrates that the HFpEF-ABA score captures the integrated biological consequences of risk factors (the actual end-organ damage) rather than merely the risk factors themselves. These structural associations persisted after full adjustment for age, sex, BMI, and comorbidities, confirming independence from the age differential between risk groups.

### Prospective outcomes: validating clinical prognostic utility

Long-term prediction and specificity. The robust prediction of incident heart failure hospitalization over a median follow-up of 14.94 years serves as the ultimate clinical validation. This extended latency establishes genuine predictive utility. Although the end-point captures all-cause heart failure, the specific context, where high-risk individuals predominantly exhibited preserved ejection fraction and concentric remodelling, strongly suggests the predicted events are mechanistically consistent with HFpEF development. Crucially, the score demonstrated high specificity: the risk for heart failure (HR 6.44) was disproportionately higher than for all-cause mortality (HR 2.81). This divergence indicates that the score captures a specific cardiac vulnerability rather than generic frailty or diffuse health decline.

The continuum of risk. The continuous log-linear associations confirm that risk operates on a biological continuum without threshold effects. This implies that the underlying drivers (metabolic inflammation, senescence) accumulate progressively.^29,30^ Clinically, this supports treating this susceptibility as a spectrum disorder analogous to pre-diabetes, suggesting that preventive interventions should be graded according to continuous risk rather than binary cut-offs.

Subgroup insights. Unmasking the ‘pure’ signal prognostic signals were most potent in younger participants and those without established comorbidities. This observation provides critical mechanistic insight. In older or multimorbid cohorts, the specific HFpEF signal is often diluted by competing risks (e.g. ischaemic events) or attenuated by concurrent cardio-protective therapies.^31^ Conversely, in younger and ‘healthier’ phenotypes, the score effectively isolates early high-risk cardiac remodelling patterns before it is obscured by clinical noise. This suggests the score offers the greatest incremental value precisely where traditional clinical assessment often underestimates risk.

### Integrating structure and outcomes: inferring disease trajectories

Our study integrates structural phenotyping with prospective risk assessment to reconstruct the longitudinal pathophysiology of HFpEF. The constituent elements of the score (age, BMI, AF) are established mechanistic drivers: advanced age propels myocardial fibrosis and arterial stiffening. Importantly, obesity is increasingly recognized not merely as a comorbidity, but as a central pathophysiological driver of the HFpEF phenotype. Recent work proposing the ‘adipokine hypothesis’ of HFpEF suggests that excess adipose tissue and dysregulated adipokine signalling contribute to systemic inflammation, endothelial dysfunction, myocardial fibrosis, and adverse cardiac remodelling, thereby promoting progression toward overt HFpEF.^32^ Atrial fibrillation both results from and accelerates atrial myopathy.^33^ The strong association observed between higher scores and adverse remodelling (LA dysfunction, concentric hypertrophy) confirms that the score effectively captures the cumulative biological impact of these upstream risk factors.

Crucially, the prospective outcome data provide the temporal link between this anatomical substrate and clinical prognosis. The 6.4-fold excess risk of heart failure hospitalization over 14.94 years validates that the structural phenotypes identified by the score are not benign bystanders but represent clinically significant pathology. Furthermore, the magnitude of remodelling implies chronicity; the 7% absolute reduction in LAEF matches changes seen in symptomatic HFpEF, suggesting that high-risk individuals have experienced a prolonged period of subclinical haemodynamic stress.^34^ Collectively, these findings support a continuum of risk characterized by progressive cardiac remodelling preceding overt heart failure: the score identifies individuals with measurable structural damage that serves as the precursor to clinical decompensation, offering a prime window for preventive intervention.

### Therapeutic implications: early risk stratification and prevention of heart failure

Targeting the preclinical substrate with phenotype-specific therapy. The identification of reversible structural abnormalities (LA dysfunction, concentric remodelling) in high-risk individuals provides the biological rationale for early intervention. This supports deploying therapies proven to modify this specific metabolic-inflammatory substrate. SGLT2 inhibitors, which reduced heart failure events in the DECLARE-TIMI 58 trial, likely confer benefit by stabilizing this unrecognized high-risk phenotype for heart failure.^35,36^ Similarly, since obesity is a core component of the score, GLP-1 receptor agonists offer a mechanistically precise intervention.^37,38^ These agents reverse adverse remodelling and demonstrated HF reduction in the SELECT trial, suggesting that score-guided treatment could arrest disease progression before irreversible fibrosis ensues.^39^

Implementation: a two-step strategy. The score’s reliance on routine clinical variables enables scalable automation via electronic health records. To address the modest positive predictive value inherent in general population screening, we propose a two-step strategy: using the score as a sensitive ‘gatekeeper’ to identify high-risk individuals for downstream echocardiography or natriuretic peptide testing.^40^ This approach optimizes resource allocation and creates a feasible framework for randomized preventive trials, which are urgently needed to establish early prevention strategies in high-risk individuals analogous to cardiovascular disease prevention.

### Limitations

Our study has several limitations. First, the UK Biobank cohort is predominantly White and based entirely in the United Kingdom, which may limit the generalizability of our findings to other ethnicities and healthcare systems, particularly populations with different metabolic risk profiles, such as Asian populations. In addition, the UK Biobank is subject to a ‘healthy volunteer’ bias. Second, although participants with known heart failure were excluded at baseline, the UK Biobank lacks comprehensive symptom assessment and systematic LVEF data during follow-up. Therefore, study outcomes should be interpreted as incident heart failure rather than definitively confirmed HFpEF. Although the high-risk group showed structural features consistent with HFpEF physiology, we cannot completely exclude incident HFrEF events. Third, NT-proBNP measurements were not routinely available in the cohort, limiting biomarker-based assessment. Fourth, serial CMR imaging was unavailable. Therefore, although elevated HFpEF-ABA scores were associated with adverse remodelling and subsequent heart failure events, longitudinal remodelling progression could not be directly assessed. Finally, our findings demonstrate prognostic utility rather than treatment-guiding utility. Further randomized studies are needed to determine whether score-guided interventions can prevent progression to overt heart failure.

## Conclusion

In this large-scale analysis of community participants, we validate the HFpEF-ABA score as a robust tool for identifying individuals at high risk for incident heart failure, providing dual validation for expanding its utility from diagnosis to preventive screening. Biological validation in 65 293 participants demonstrated that elevated scores strongly associate with HFpEF-specific structural hallmarks, including left atrial dysfunction and concentric remodelling, confirming the presence of measurable pathophysiological substrate years before diagnosis. Complementing this, clinical validation in 416 374 participants established a clear temporal sequence, where baseline score elevation predicted a 6.4-fold excess risk of heart failure hospitalization over 14.94 years. The convergence of subclinical phenotyping and long-term outcome prediction supports a paradigm shift: moving beyond the treatment of established failure to the prevention of heart failure progression through score-guided strategies utilizing SGLT2 inhibitors, GLP-1 receptor agonists, and intensive lifestyle modification.

## Supplementary Material

xvag171_Supplementary_Data

## Declarations

### Data Availability

The data that support the findings of this study are available from UK Biobank (https://www.ukbiobank.ac.uk/), but restrictions apply to the availability of these data, which were used under license for the current study (application 1034464), and so are not publicly available. Data are however available from the authors upon reasonable request and with permission of UK Biobank.

### Pre-registered Clinical Trial Number

None supplied.
