# Supplementary material for: The HFpEF-ABA score predicts adverse cardiac remodelling and incident heart failure: a UK biobank study
Source: ESC Heart Fail. 2026 Jul 7;13(4):xvag171. doi: 10.1093/eschf/xvag171 (PMC13340748; doi:10.1093/eschf/xvag171)
Supplement: xvag171_Supplementary_Data [file xvag171_supplementary_data.docx]

Table S1. Clinical Characteristics and Cardiac Magnetic Resonance Phenotyping of the Imaging Subcohort.

| **Characteristic** | **Overall**  **(n=65293)** | **Low (<25%)**  **(n=6493)** | **Intermediate (25-80%)**  **(n=55760)** | **High (>80%)**  **(n=3040)** | **p** |
| --- | --- | --- | --- | --- | --- |
| Age | 66.00 [60.00, 72.00] | 55.00 [53.00, 59.00] | 67.00 [61.00, 72.00] | 72.00 [67.00, 77.00] | <0.001 |
| BMI | 25.39 [23.03, 28.38] | 21.53 [20.08, 22.99] | 25.71 [23.51, 28.40] | 33.69 [27.33, 38.06] | <0.001 |
| Male sex | 29830 (45.7) | 1893 (29.2) | 26418 (47.4) | 1519 (50.0) | <0.001 |
| Ethnicity |  |  |  |  | <0.001 |
| White | 62811 (96.2) | 6150 (94.7) | 53702 (96.3) | 2959 (97.3) |  |
| Asian | 1051 (1.6) | 173 (2.7) | 860 (1.5) | 18 (0.6) |  |
| Black | 470 (0.7) | 44 (0.7) | 402 (0.7) | 24 (0.8) |  |
| Townsend | -2.57 [-3.88, -0.40] | -2.37 [-3.78, 0.00] | -2.60 [-3.90, -0.46] | -2.44 [-3.79, -0.06] | <0.001 |
| Education  (College Degree) | 9585 (14.7) | 1056 (16.3) | 8139 (14.6) | 390 (12.8) | <0.001 |
| Smoking |  |  |  |  | <0.001 |
| Never | 41002 (62.8) | 4662 (71.8) | 34647 (62.2) | 1693 (55.7) |  |
| Previous | 22094 (33.8) | 1526 (23.5) | 19302 (34.6) | 1266 (41.7) |  |
| Current | 2178 (3.3) | 304 (4.7) | 1795 (3.2) | 79 (2.6) |  |
| Alcohol consumption |  |  |  |  |  |
| Never | 2225 (3.4) | 216 (3.3) | 1856 (3.3) | 153 (5.0) | <0.001 |
| Previous | 2465 (3.8) | 227 (3.5) | 2067 (3.7) | 171 (5.6) |  |
| Current | 60600 (92.8) | 6050 (93.2) | 51834 (93.0) | 2716 (89.3) |  |
| Hypertension | 17007 (26.0) | 432 (6.7) | 14869 (26.7) | 1706 (56.1) | <0.001 |
| Diabetes mellitus | 3292 (5.0) | 79 (1.2) | 2811 (5.0) | 402 (13.2) | <0.001 |
| Coronary artery disease | 3067 (4.7) | 54 (0.8) | 2601 (4.7) | 412 (13.6) | <0.001 |
| Atrial fibrillation | 1462 (2.2) | 0 (0.0) | 157 (0.3) | 1305 (42.9) | <0.001 |
| LVEF (median [IQR]) | 60.38 [56.69, 64.21] | 60.12 [56.66, 63.77] | 60.39 [56.70, 64.24] | 60.64 [56.63, 64.67] | <0.001 |
| LVMi (median [IQR]) | 43.74 [38.78, 49.80] | 42.14 [37.93, 47.76] | 43.91 [38.89, 49.97] | 44.14 [38.96, 50.25] | <0.001 |
| LVEDVi (median [IQR]) | 75.80 [67.48, 85.06] | 80.69 [72.97, 89.94] | 75.40 [67.25, 84.65] | 71.66 [62.88, 81.54] | <0.001 |
| LVESVi (median [IQR]) | 29.74 [25.19, 35.03] | 31.84 [27.70, 37.03] | 29.59 [25.05, 34.87] | 27.91 [23.24, 33.34] | <0.001 |
| LVSVi (median [IQR]) | 45.75 [40.66, 51.51] | 48.65 [43.30, 54.47] | 45.56 [40.52, 51.24] | 43.47 [38.18, 49.63] | <0.001 |
| GLS (median [IQR]) | -18.64 [-20.36, -17.00] | -18.32 [-20.05, -16.77] | -18.68 [-20.39, -17.05] | -18.51 [-20.47, -16.46] | <0.001 |
| LAEF (median [IQR]) | 60.92 [55.86, 66.15] | 61.92 [57.68, 66.39] | 61.05 [56.02, 66.32] | 55.48 [47.73, 61.48] | <0.001 |
| LAVi (median [IQR]) | 37.70 [30.97, 45.15] | 39.05 [32.87, 45.73] | 37.37 [30.64, 44.75] | 41.83 [33.37, 52.05] | <0.001 |
| RVEF (median [IQR]) | 57.92 [54.08, 61.67] | 57.93 [54.29, 61.52] | 57.96 [54.13, 61.71] | 57.24 [52.79, 61.43] | <0.001 |
| RVEDVi (median [IQR]) | 80.66 [71.47, 91.31] | 84.88 [75.31, 95.74] | 80.44 [71.32, 91.01] | 76.95 [67.35, 87.39] | <0.001 |
| RVESVi (median [IQR]) | 33.88 [28.41, 40.20] | 35.63 [30.03, 42.13] | 33.74 [28.30, 40.04] | 32.98 [27.32, 39.17] | <0.001 |
| RVSVi (median [IQR]) | 46.77 [41.22, 52.80] | 49.12 [43.80, 55.21] | 46.67 [41.17, 52.63] | 43.80 [37.90, 50.23] | <0.001 |
| RAEF (median [IQR]) | 47.02 [41.42, 52.87] | 47.78 [42.45, 53.19] | 46.97 [41.41, 52.83] | 46.26 [38.75, 52.98] | <0.001 |
| RAVi (median [IQR]) | 44.22 [36.48, 53.21] | 47.41 [40.32, 55.91] | 43.93 [36.26, 52.84] | 42.03 [33.24, 53.71] | <0.001 |

Abbreviations: CMR, cardiac magnetic resonance; LVEF, left ventricular ejection fraction; LVMi, left ventricular mass index; LVEDVi, left ventricular end-diastolic volume index; LVESVi, left ventricular end-systolic volume index; LVSVi, left ventricular stroke volume index; GLS, global longitudinal strain; LAEF, left atrial ejection fraction; LAVi, left atrial volume index; RVEF, right ventricular ejection fraction; RVEDVi, right ventricular end-diastolic volume index; RVESVi, right ventricular end-systolic volume index; RAEF, right atrial ejection fraction; RAVi, right atrial volume index.

Table S2. Multivariable linear regression of CMR parameters by HFpEF-ABA risk category in the imaging subcohort

| **CMR Parameter** | **Risk Group** | **Age + Sex Adjusted β (95% CI)** | **Fully Adjusted† β (95% CI)** |
| --- | --- | --- | --- |
| LA Ejection Fraction (%) | Intermediate Risk | 1.30 (1.03, 1.57)* | 1.52 (1.24, 1.80)* |
|  | High Risk | −5.10 (−5.60, −4.60)* | −4.61 (−5.10, −4.11)* |
| LA Volume Index (ml/m²) | Intermediate Risk | −0.75 (−1.11, −0.40)* | −0.90 (−1.25, −0.54)* |
|  | High Risk | 5.89 (5.24, 6.53)* | 5.39 (4.76, 6.02)* |
| LV Mass Index (g/m²) | Intermediate Risk | 0.18 (−0.01, 0.38) | 0.30 (0.11, 0.49)* |
|  | High Risk | 0.28 (−0.07, 0.63) | 0.36 (0.01, 0.71)* |
| LV End-Diastolic Volume Index (ml/m²) | Intermediate Risk | −0.52 (−0.89, −0.16)* | −0.29 (−0.66, 0.07) |
|  | High Risk | 1.94 (1.26, 2.62)* | 2.35 (1.68, 3.02)* |
| LV Ejection Fraction (%) | Intermediate Risk | −0.15 (−0.30, 0.01) | −0.02 (−0.17, 0.13) |
|  | High Risk | −0.27 (−0.54, −0.00)* | −0.13 (−0.40, 0.14) |
| Global Longitudinal Strain (%) | Intermediate Risk | −0.26 (−0.34, −0.18)* | −0.21 (−0.29, −0.13)* |
|  | High Risk | 0.67 (0.52, 0.82)* | 0.54 (0.39, 0.69)* |
| RA Volume Index (ml/m²) | Intermediate Risk | 0.72 (0.32, 1.12)* | 0.92 (0.52, 1.32)* |
|  | High Risk | 9.54 (8.82, 10.26)* | 9.02 (8.31, 9.73)* |
| RV Ejection Fraction (%) | Intermediate Risk | 0.22 (0.05, 0.39)* | 0.28 (0.11, 0.45)* |
|  | High Risk | −0.72 (−1.03, −0.42)* | −0.66 (−0.96, −0.36)* |

Reference category: Low Risk (<25%). * P < 0.05.

† Fully Adjusted: adjusted for age, sex, BMI, hypertension, diabetes mellitus, and coronary artery disease.

Abbreviations: CMR, cardiac magnetic resonance; LA, left atrial; LV, left ventricular; RA, right atrial; RV, right ventricular; β, regression coefficient; CI, confidence interval.

Table S3. Association between HFpEF-ABA risk categories and adverse clinical outcomes.

| **Outcome** | **Group** | **Event_n_N** | **Model 1 HR**  **(95% CI)** | **P Model 1** | **Model 2 HR**  **(95% CI)** | **P Model 2** | **Model 3 HR**  **(95% CI)** | **P Model 3** |
| --- | --- | --- | --- | --- | --- | --- | --- | --- |
| Composite Outcome | High (>80%) | 3,457 / 10,646 (32.5%) | 8.17 (7.80-8.56) | <0.001 | 8.09 (7.73-8.48) | <0.001 | 3.46 (3.29-3.63) | <0.001 |
| Composite Outcome | Intermediate (25-80%) | 37,864 / 298,264 (12.7%) | 3.05 (2.95-3.15) | <0.001 | 3.06 (2.96-3.16) | <0.001 | 2.04 (1.97-2.11) | <0.001 |
| Composite Outcome | Low (<25%) | 3,951 / 107,464 (3.7%) | 1.00 (Ref) |  | 1.00 (Ref) |  | 1.00 (Ref) |  |
| HF Hospitalization | High (>80%) | 1,722 / 10,646 (16.2%) | 21.71 (19.88-23.71) | <0.001 | 21.19 (19.39-23.15) | <0.001 | 6.44 (5.86-7.08) | <0.001 |
| HF Hospitalization | Intermediate (25-80%) | 10,881 / 298,264 (3.6%) | 4.71 (4.36-5.08) | <0.001 | 4.70 (4.36-5.07) | <0.001 | 2.65 (2.45-2.87) | <0.001 |
| HF Hospitalization | Low (<25%) | 716 / 107,464 (0.7%) | 1.00 (Ref) |  | 1.00 (Ref) |  | 1.00 (Ref) |  |
| All-Cause Mortality | High (>80%) | 2,567 / 10,646 (24.1%) | 6.43 (6.10-6.77) | <0.001 | 6.38 (6.06-6.72) | <0.001 | 2.81 (2.66-2.98) | <0.001 |
| All-Cause Mortality | Intermediate (25-80%) | 31,236 / 298,264 (10.5%) | 2.84 (2.74-2.95) | <0.001 | 2.86 (2.76-2.96) | <0.001 | 1.93 (1.86-2.01) | <0.001 |
| All-Cause Mortality | Low (<25%) | 3,447 / 107,464 (3.2%) | 1.00 (Ref) |  | 1.00 (Ref) |  | 1.00 (Ref) |  |

Data are presented as hazard ratios (95% confidence intervals).

Model 1: Adjusted for sex, ethnicity, education, and Townsend deprivation index.

Model 2: Adjusted for Model 1 plus smoking, alcohol status, and physical activity.

Model 3: Adjusted for Model 2 plus hypertension, diabetes, CAD, lipid-lowering medication, and eGFR.

Abbreviations: CI, confidence interval; HR, hazard ratio; Ref, reference.

Table S4. Sensitivity analysis excluding participants with events or follow-up less than 2 years (Landmark Analysis).

| **Outcome** | **Group** | **Event_n_N** | **Model 1 HR**  **(95% CI)** | **P Model 1** | **Model 2 HR**  **(95% CI)** | **P Model 2** | **Model 3 HR**  **(95% CI)** | **P Model 3** |
| --- | --- | --- | --- | --- | --- | --- | --- | --- |
| Composite Outcome | High (>80%) | 3,251 / 10,440 (31.1%) | 8.23 (7.84-8.63) | <0.001 | 8.15 (7.77-8.55) | <0.001 | 3.52 (3.34-3.70) | <0.001 |
| Composite Outcome | Intermediate (25-80%) | 36,375 / 296,775 (12.3%) | 3.11 (3.01-3.22) | <0.001 | 3.12 (3.02-3.23) | <0.001 | 2.09 (2.02-2.17) | <0.001 |
| Composite Outcome | Low (<25%) | 3,731 / 107,244 (3.5%) | 1.00 (Ref) |  | 1.00 (Ref) |  | 1.00 (Ref) |  |
| HF Hospitalization | High (>80%) | 1,595 / 10,440 (15.3%) | 21.31 (19.46-23.34) | <0.001 | 20.82 (19.01-22.81) | <0.001 | 6.45 (5.86-7.11) | <0.001 |
| HF Hospitalization | Intermediate (25-80%) | 10,404 / 296,775 (3.5%) | 4.74 (4.38-5.12) | <0.001 | 4.74 (4.38-5.12) | <0.001 | 2.70 (2.49-2.92) | <0.001 |
| HF Hospitalization | Low (<25%) | 682 / 107,244 (0.6%) | 1.00 (Ref) |  | 1.00 (Ref) |  | 1.00 (Ref) |  |
| All-Cause Mortality | High (>80%) | 2,406 / 10,440 (23%) | 6.50 (6.16-6.86) | <0.001 | 6.47 (6.13-6.82) | <0.001 | 2.88 (2.72-3.05) | <0.001 |
| All-Cause Mortality | Intermediate (25-80%) | 29,990 / 296,775 (10.1%) | 2.91 (2.81-3.02) | <0.001 | 2.93 (2.82-3.04) | <0.001 | 1.99 (1.91-2.06) | <0.001 |
| All-Cause Mortality | Low (<25%) | 3,248 / 107,244 (3%) | 1.00 (Ref) |  | 1.00 (Ref) |  | 1.00 (Ref) |  |

Footnotes:

This analysis excluded events occurring within the first 2 years of follow-up to minimize reverse causality.

Models 1–3 were adjusted for the same covariates as in the main analysis (Table 2).

Abbreviations: CI, confidence interval; HR, hazard ratio; Ref, reference.

Table S5. Subgroup analysis of the association between high HFpEF-ABA risk and the primary composite outcome.

| Subgroup | Level | Event_n_N | HR (95% CI) | P for Interaction |
| --- | --- | --- | --- | --- |
| Age | < 60 Years | 12,357/235,882 (5.2%) | 2.58 (2.34-2.86) | < 0.001 |
| Age | >= 60 Years | 32,915/180,492 (18.2%) | 1.41 (1.29-1.55) |  |
| Sex | Male | 27,243/193,024 (14.1%) | 3.54 (3.31-3.79) | 0.787 |
| Sex | Female | 18,029/223,350 (8.1%) | 3.32 (3.08-3.58) |  |
| BMI | Non-Obese (<30) | 30,336/315,784 (9.6%) | 4.03 (3.71-4.37) | 0.283 |
| BMI | Obese (>=30) | 14,936/100,590 (14.8%) | 5.17 (2.58-10.37) |  |
| Hypertension | No Hypertension | 25,267/305,812 (8.3%) | 3.61 (3.35-3.90) | < 0.001 |
| Hypertension | Hypertension | 20,005/110,562 (18.1%) | 2.64 (2.42-2.88) |  |
| Diabetes | No Diabetes | 39,625/395,923 (10%) | 3.66 (3.46-3.86) | < 0.001 |
| Diabetes | Diabetes | 5,647/ 20,451 (27.6%) | 2.10 (1.75-2.53) |  |
| Smoking | Non/Ex-Smoker | 37,078/372,480 (10%) | 4.48 (4.24-4.75) | < 0.001 |
| Smoking | Current Smoker | 8,194/ 43,894 (18.7%) | 1.82 (1.60-2.08) |  |
| Renal Function | No CKD | 42,196/407,719 (10.3%) | 4.86 (4.62-5.11) | < 0.001 |
| Renal Function | CKD (eGFR < 60) | 3,076/ 8,655 (35.5%) | 1.91 (1.47-2.47) |  |
| CAD History | No CAD | 39,063/396,827 (9.8%) | 3.57 (3.38-3.77) | < 0.001 |
| CAD History | History of CAD | 6,209/ 19,547 (31.8%) | 2.09 (1.76-2.49) |  |

Footnotes:

Hazard ratios (HR) compare the High Risk group (>80%) with the Low Risk group (<25%).All analyses were adjusted for the full set of covariates (Model 3), except for the stratification variable itself.P for Interaction indicates the statistical significance of the interaction between the risk group and the subgroup variable.Abbreviations: CAD, coronary artery disease; CKD, chronic kidney disease; CI, confidence interval; HR, hazard ratio.

Table S6. Incremental predictive value of the HFpEF-ABA score beyond age alone.

| Outcome | C-index (Age only) | C-index (ABA Score) | ΔC-index (P value) | IDI % (95% CI) | P (IDI) | NRI % (95% CI) | P (NRI) |
| --- | --- | --- | --- | --- | --- | --- | --- |
| HF Hospitalization | 0.730 | 0.749 | +0.019 (<0.001) | 0.37 (0.11–0.62) | <0.001 | 32.26 (−1.00–65.51) | <0.001 |
| Composite Outcome | 0.707 | 0.719 | +0.012 (<0.001) | 0.18 (0.12–0.23) | 0.020 | 12.49 (2.50–22.49) | <0.001 |
| All-Cause Mortality | 0.708 | 0.714 | +0.006 (<0.001) | 0.01 (−0.12–0.14) | 0.812 | 3.50 (−3.04–10.03) | 0.337 |

C-index comparison performed using z-tests. IDI and NRI calculated at the 5-year horizon (survIDINRI package), comparing an age-only baseline model vs. age + HFpEF-ABA probability. Conducted as sensitivity analyses in a random subsample of 10,000 participants (seed = 2024, 100 perturbation iterations).

Abbreviations: ABA, Age-BMI-AF; IDI, Integrated Discrimination Improvement; NRI, Net Reclassification Improvement; HF, heart failure.


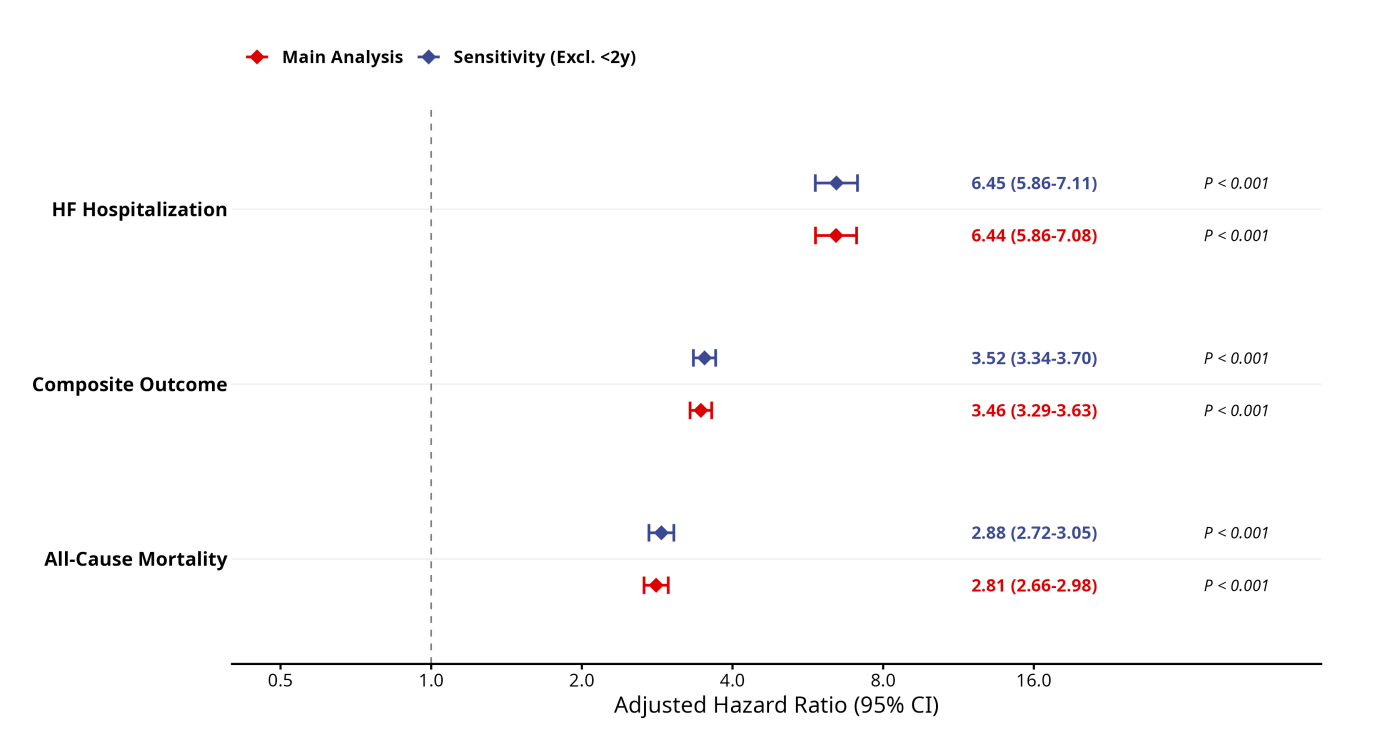


Figure S1. Sensitivity analysis excluding events within the first 2 years.

Legend:

Comparison of adjusted hazard ratios between the Main Analysis (red) and the Landmark Analysis excluding the first 2 years of follow-up (blue).


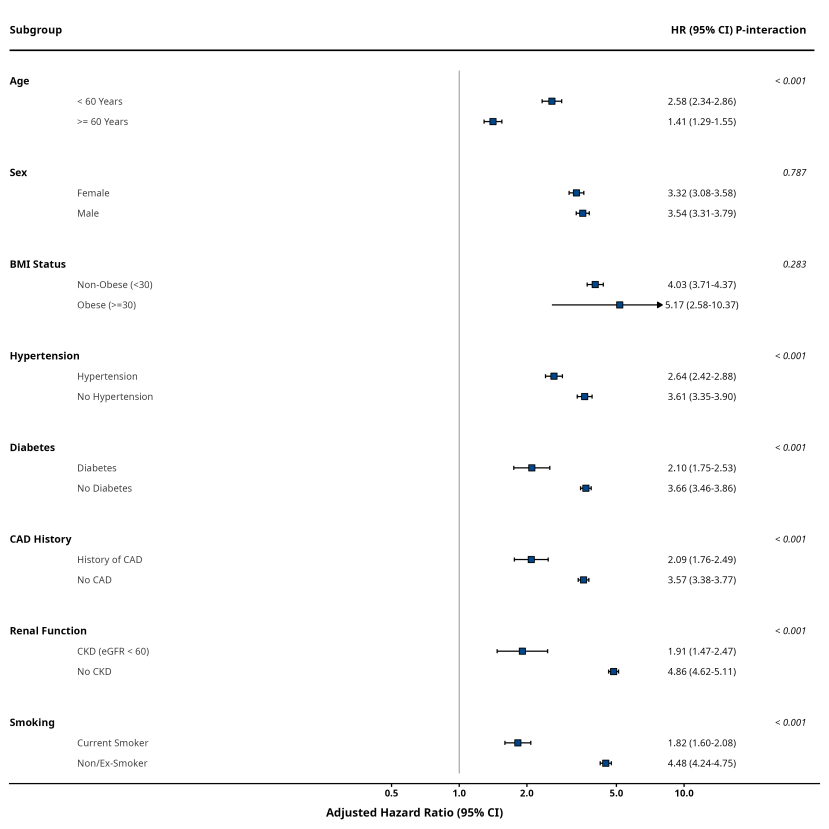


Figure S2. Subgroup analysis for the primary composite outcome.

Legend:

Adjusted hazard ratios (HR) for the High Risk group (vs. Low Risk) across prespecified subgroups. P values indicate the significance of interaction terms.


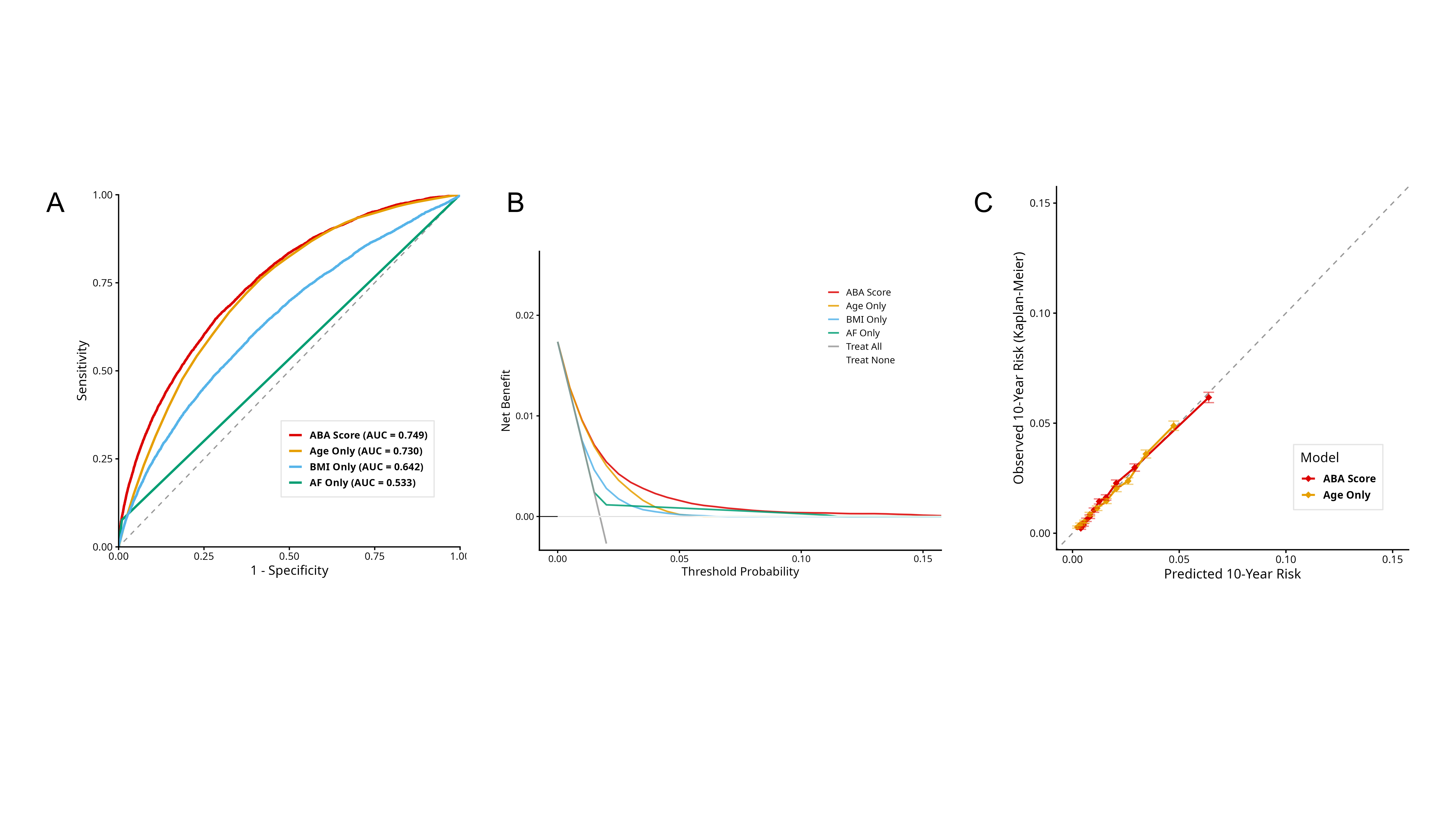


Figure S3. Diagnostic and predictive performance of the HFpEF-ABA score.

Legend:

(A) Time-dependent ROC curves for 10-year HF hospitalization risk compared with single risk factors.

(B) Decision curve analysis (DCA) showing the net benefit of the ABA score.

(C) Calibration plot comparing predicted vs. observed 10-year risk probabilities.
